# Mechanisms of Lung Damage and Development of COPD Due to Household Biomass-Smoke Exposure: Inflammation, Oxidative Stress, MicroRNAs, and Gene Polymorphisms

**DOI:** 10.3390/cells12010067

**Published:** 2022-12-23

**Authors:** Blanca Ortiz-Quintero, Israel Martínez-Espinosa, Rogelio Pérez-Padilla

**Affiliations:** 1 Research Unit, Department of Research in Biochemistry, Instituto Nacional de Enfermedades Respiratorias Ismael Cosío Villegas, Mexico City 14080, Mexico; 2 Department of Research in Tobacco and Chronic Obstructive Pulmonary Disease (COPD), Instituto Nacional de Enfermedades Respiratorias Ismael Cosío Villegas, Mexico City 14080, Mexico

**Keywords:** biomass smoke, woodsmoke, household air pollution, lung damage, COPD, pathogenesis mechanisms

## Abstract

Chronic exposure to indoor biomass smoke from the combustion of solid organic fuels is a major cause of disease burden worldwide. Almost 3 billion people use solid fuels such as wood, charcoal, and crop residues for indoor cooking and heating, accounting for approximately 50% of all households and 90% of rural households globally. Biomass smoke contains many hazardous pollutants, resulting in household air pollution (HAP) exposure that often exceeds international standards. Long-term biomass-smoke exposure is associated with Chronic Obstructive Pulmonary Disease (COPD) in adults, a leading cause of morbidity and mortality worldwide, chronic bronchitis, and other lung conditions. Biomass smoke-associated COPD differs from the best-known cigarette smoke-induced COPD in several aspects, such as a slower decline in lung function, greater airway involvement, and less emphysema, which suggests a different phenotype and pathophysiology. Despite the high burden of biomass-associated COPD, the molecular, genetic, and epigenetic mechanisms underlying its pathogenesis are poorly understood. This review describes the pathogenic mechanisms potentially involved in lung damage, the development of COPD associated with wood-derived smoke exposure, and the influence of genetic and epigenetic factors on the development of this disease.

## 1. Introduction

Nearly 3 billion people worldwide use solid organic materials as the primary fuel for indoor cooking and heating, mainly in developing countries, resulting in biomass smoke and household air pollution (HAP) [1,2]. Biomass smoke is generated from the incomplete combustion of organic material such as wood, crop residues, twigs, dried animal dung, and charcoal in rudimentary stoves or open fires. This biomass smoke contains high concentrations of hazardous inhalable particulate matter [3] and chemical volatiles, such as carbon monoxide, sulfurs, and polycyclic aromatic hydrocarbons, which are harmful to human health [4,5,6]. According to the World Health Organization (WHO), over 3.8 million people die prematurely every year from diseases attributable to HAP from using organic material as fuel for cooking. These deaths are due to childhood and adult pneumonia (27%), ischemic heart disease (27%), chronic obstructive pulmonary disease (COPD) (20%), strokes (18%), and lung cancer (8%) [1].

In 2010, the American Thoracic Society (ATS) issued an official statement including indoor biomass smoke as a likely new risk factor for COPD [7]. Further accumulative evidence confirmed that chronic exposure to indoor biomass smoke was an independent risk factor for COPD, with woodsmoke being the most important among the indoor biomass smoke from fuels [8].

COPD is a complex and heterogeneous disease characterized by an atypical inflammatory response to chronic exposure to inhaled toxic particles, causing damage to lung tissue and in susceptible individuals [9]. The disease shows high heterogeneity in terms of pulmonary pathology, immune response, clinical features, and phenotype. Affected lungs exhibit destruction of the parenchyma, emphysema, mucus hypersecretion, airway thickening, scarring, focal fibrosis, and loss of the small airways [9,10].

Cigarette smoking is the main risk factor for COPD. Most knowledge about the clinical symptoms, phenotype, physiology, pathology, and treatment has been obtained from studies on smoking-related COPD patients. However, 25–45% of COPD patients are nonsmokers [11,12], with biomass-smoke exposure being an important risk factor (OR: 2.44) [13], and pooled estimates by fuel type showed that woodsmoke (OR: 4.29) is the most important risk factor for COPD [14]. Several studies have shown that, compared to smoking-related COPD, biomass-related COPD exhibits a predominant airway involvement, with higher bronchial hyperreactivity and bronchodilator response. It predominantly affects small airways with less emphysema and causes a slower rate of decline in lung function [15,16,17]. These findings suggest that biomass smoke-related lung damage and COPD may involve different mechanisms of lung pathophysiology, which have been poorly studied. Although biomass smoke contains high concentrations of particulate matter and chemical compounds that are as hazardous as cigarette smoke, the exposure to pollutants may be milder in women exposed to biomass smoke while cooking due to predominant nose inhalation and normal flow rates compared with tobacco smoking, for which the inhalation pattern involves higher flow rates through the mouth [18]. Importantly, in addition to exposure to environmental risk factors, the development of COPD depends on genetic susceptibility, epigenetic genetic regulation, and other intrinsic factors that have been better studied in smoking-related COPD but little investigated in biomass-related COPD.

A better understanding of the molecular mechanisms underlying biomass-induced COPD and the genetic and epigenetic factors influencing COPD outcomes will provide insights into pathophysiology and disease progression that may guide the development of phenotype-targeted treatment.

In this article, we describe the currently available evidence for the mechanisms of lung damage due to biomass exposure and those potentially involved in biomass-related COPD related to household wood burning for cooking and heating, which is the leading risk factor among the use of different fuel types that produce indoor biomass smoke. We highlight the evidence for the influence of genetic and epigenetic factors on developing this disease phenotype.

## 2. Components of Woodsmoke Pollutants

Wood burning generates inefficient and incomplete combustion with higher emissions of harmful chemicals and fine particulate matter compared to burning more efficient fuels, such as liquefied petroleum gas (LPG) [3,19].

Woodsmoke components include a complex mixture of hundreds of chemical volatiles with toxic, carcinogenic, and irritant properties and respirable particulate matter (PM) that can penetrate the airways and the lungs, many shared by tobacco smoke. Each component can be present in the gas phase, particle phase, or both phases, depending on its volatility [20]. Although the exact composition of the volatile chemicals and PM varies according to the specific wood type and combustion conditions, its major harmful components include carbon monoxide (CO), benzene, aldehydes, polycyclic aromatic hydrocarbons (PAHs), nitrogen oxides (such as NO_2_), sulfur oxides (such as SO_2_), free radicals, and fine particulate matter with a diameter less than 10 μm (PM_10_) [20,21,22]. A résumé of relevant health-hazardous volatile chemicals and particulate matter present in woodsmoke is listed in Table 1. Because the effect of biomass and cigarette smoke exposure are compared in many of the published cohort studies [21,23,24,25,26], Supplementary Table S1 compares reported pollutant chemicals and PM components of wood and cigarette smoke [27,28].

### 2.1. Particulate Matter (PM)

PM is classified according to the mass median aerodynamic diameter into coarse particles with a diameter of 2.5–10 μm (PM_10_), fine particles with a diameter < 2.5 μm (PM_2.5_), and ultrafine particles (UFP) with a diameter < 0.1 μm (PM_0.1_). The primary source of PM_2.5_ and PM_0.1_ are emissions from the combustion of vehicle fuels (gasoline, diesel), wood, and coal. Meanwhile, PM_10_ particles are derived predominantly from abraded soil, road dust, construction debris, and oil combustion products with bioaerosols. Importantly, PM varies in size, shape, and chemical composition depending on the source, which includes inorganic ions (e.g., chloride, nitrates, ammonium, and sulfates), heavy metals (e.g., cadmium, copper, and zinc), black carbon, and organic compounds (e.g., phenol, and PAHs) [29,30]. Therefore, the interpretation of the biological effects and health impact of PM depends on the diverse chemical and physical characteristics, sources, and the potential dynamic changes of PM suspended in the air [29]. Nevertheless, the general focus of the impact of ambient PM on health outcomes has been on particles with aerodynamic diameters less than or equal to 2.5 or 10 μm (PM_2.5_ and PM_10_) [29]. Both PM_2.5_ and PM_10_ are associated with increased mortality from all causes, and specific causes include cardiovascular disease, respiratory disease (asthma and COPD), and lung cancer [25,31,32]. Exposure to PM from woodsmoke is toxic to humans, animals, and in vitro models, due to several mechanisms, such as airway inflammation, exacerbations of respiratory symptoms, lung inflammation, cell toxicity, and mucin expression [33,34,35]. Woodsmoke particulate matter’s physicochemical and toxicological properties depend on wood species and combustion conditions [36,37].

Burning biomass fuels in cookstoves or open fires in poorly ventilated spaces produces very high concentrations of PM_10_ and PM_2.5_ in the range of 300–3000 μg/m^3^, which can reach 30,000 μg/m^3^ during periods of cooking [3,5,38,39]. These levels exceed the WHO’s recommendations in terms of the annual mean air quality guideline (AQG) levels of 15 and 5 μg/m^3^ for PM_10_ and PM_2.5_, respectively [29], with interim target levels of 70 and 35 μg/m^3^ for PM_10_ and PM_2.5_, respectively. However, studies indicate that most woodsmoke particles are smaller than 1 μm (UFP or PM_0.1_), with a peak in diameter distribution between 0.15 and 0.4 μm [40,41], which is in the inhalable size range and effect on lung inflammation and cell toxicity [25,36,37,42,43]. Therefore, woodsmoke is a dangerous combination of highly concentrated chemical components with toxic and carcinogenic properties and fine inhalable particles that can penetrate the lungs.

### 2.2. Woodsmoke Pattern of Particulate Matter Deposition in the Lungs

Using high-fidelity computational fluid-particle dynamic (CFPD) simulations of airflow and particle deposition analysis [18], a study showed that the concentrations of cigarette smoke inhaled are three orders of magnitude higher than those of biomass smoke in the lung. In addition, cigarette smoke has higher PM intrathoracic deposition in the lungs and higher inhalation flow rates than biomass smoke. Biomass smoke consisted of a bimodal log-normal distribution with PM_50–0.05_, while cigarette smoke consisted mostly of PM_1–0.1_. Higher fractions of cigarette smoke were in the last few airway generations compared to biomass smoke due to the higher flow rates. Therefore, biomass smoke nasally inhaled at low flow rates at an average tidal volume likely prevents smoke penetration beyond the small airways such that biomass smoke results in an airway-dominant COPD phenotype with less emphysema [44,45].

## 3. Biomass-Smoke Exposure Burden and Lung Disease Outcomes

The household use of solid fuels for cooking and heating, primarily wood, is the most widespread source of indoor air pollution worldwide. About 2.8 billion people—36% of the world’s population—use solid fuels as their primary energy source [46]. Populations in less developed countries and rural areas are most exposed to indoor air pollution due to solid fuel use. On a global scale, the proportion of people mainly using polluting fuels for cooking is 61% in rural areas and less than 20% in urban areas. Most exposed people live in sub-Saharan Africa, with about 84% of exposed individuals, and Central Asia and South Asia, with approximately 30%. In contrast, the proportion of people using solid fuel in developed countries, such as those in North America and Europe, is about 6% [46].

Several meta-analyses support the association of household air pollution secondary to biomass smoke with lung disease outcomes, including COPD [6,8,13,14]. Two health outcomes have strong evidence of association with indoor biomass-smoke exposure: acute lower respiratory infections (ALRIs) in children under five, and COPD in men and women 30 or over, whereas lung cancer has been associated mostly with burning coal for those aged 30 or over [6]. The overall risk of COPD in women exposed to indoor air pollution from the combustion of biomass such as wood was estimated at 3.2 (95% CI: 2.3–4.8), and that for men was 1.8 (95% CI: 1.0–3.2) [6]. Two major meta-analyses published in 2010 reported that exposure to the products of biomass combustion for domestic purposes is an independent risk factor for COPD, with an odds ratio (OR) of 2.44 (95% CI: 1.9–3.33) for developing COPD [13] in men (OR: 4.30; 95% CI: 1.85–10.01) and women (OR: 2.73; 95% CI: 2.28–3.28), and in the Asian (OR: 2.31; 95% CI: 1.41–3.78) and non-Asian populations (OR: 2.56; 95% CI: 1.71–3.83) [13]. According to another pooled estimate, woodsmoke exposure could be the most important risk factor for COPD (OR: 4.29; 95% CI: 1.35–13.70) [14]. A more recent meta-analysis from population-based studies [8] confirmed that indoor biomass exposure was among the five main risk factors for COPD globally, although the magnitude of the OR (1.4; 95% CI: 1.2–1.7) was considerably lower than that in previous studies. Two large prospective cross-sectional observational studies reported that biomass smoke [47] and particulate matter with a diameter less than 2.5 μm [48] were major risk factors for COPD.

Exposure to biomass smoke is strongly associated with poverty, also a risk factor for several health issues, including COPD. Epidemiological studies often lack quantitative exposure measurements, and observational studies cannot separate poverty from exposure to biomass smoke. Interventional studies in which households or communities are randomized into a strategy to reduce indoor pollution may provide solid information but are unacceptable to maintain for a long time in a community without improving the interventions. In addition, developing lung diseases such as COPD takes decades [49]. Most published studies rely on a questionnaire to assess exposure to household air pollution, asking about the type of fuel used, possibly ventilation, but lacking estimates of the dose inhaled or toxins reaching target organs and tissue. Studies of tobacco smoking-related COPD often use an estimate of lifetime cigarettes smoked called pack-years, the product of the average number of cigarettes smoked per day divided by 20 cigarettes per pack and years of smoking to include individuals with at least 10 pack-years. Similarly, the total duration of exposure to biomass smoke or household air pollution from solid fuels can be estimated from hour-years of exposure, the product of the years of exposure, and the number of hours per day using biomass as fuel during cooking. Ten years of exposure to HAP or >60 hour-years may be relevant for COPD, although the development of the disease depends not only on exposure but also on susceptibility; hence sharp cutoffs are arbitrary and used mainly for classification purposes. Contrasting to pack-years, hour-years of biomass-smoke exposure contain no measured biomass burned, just time, and assume a virtually uniform concentration of pollutants. Pack-years and hour-years include years of exposure and are used as simple cumulative exposure indicators. It is important to remember that more years of exposure means older age, which is associated with an increasing prevalence of COPD and other chronic diseases.

### 3.1. Developed Countries

Most studies on household air pollution secondary to biomass smoke and COPD are reported in low- and middle-income developing countries. However, according to surveillance and census data, wood is also used as fuel for household heating in developed countries such as Canada, Australia, and the US [4,46,50]. Nevertheless, different from developing countries, the use of wood as fuel is mainly seasonal during winter and with milder exposure levels due to improved stove efficiency and adequate venting in developed countries [50].

Most studies in developed countries refer to outdoor environmental pollution indirectly attributed to seasonal wood burning and the effects on respiratory symptoms, lung function, and asthma exacerbations in several parts of the US, Canada, Denmark, Australia, and New Zealand [4]. One study analyzed the indoor exposure of PM_10_ from woodstoves and respiratory health in Navajo children in Arizona [51]. Additionally, only one case–control study in Spain found that self-reported combined wood and charcoal smoke exposure was associated with a 4.5-fold greater OR of having COPD [52]. The lack of more studies indicates the underestimation of the relevance of indoor biomass exposure and COPD and lung diseases in developed countries.

### 3.2. Outdoor Air Pollution Due to Wood Burning in Wildfires

Smoke from wood burning during wildfires is a significant source of short-term exposure to high air pollution worldwide. Climate change has intensified the severity and frequency of wildfire occurrences [53,54], resulting in more people being exposed yearly. Several systematic reviews have analyzed the literature on the health effects of exposure to smoke from wildfires [4,55,56,57,58]. Most studies concluded an association between exposure, all-cause mortality, and exacerbation of respiratory symptoms in individuals with preexisting illnesses such as asthma, COPD, bronchitis, and pneumonia [55,57]. Wildfire smoke exposure association with hospital admissions for COPD among the general public was also reported in previous studies [4,59,60,61]. Adetona et al. [56] concluded that the current evidence of adverse impacts on respiratory health among the general public is strong, but regarding firefighters is weak. Wildfire smoke exposure in the general public is associated with hospital admission for COPD and asthma exacerbation, acute bronchitis, bronchiolitis, and pneumonia. Although environmental PM concentration is associated with cardiovascular morbidity and mortality [62], the authors concluded that current evidence of an association of PM exposure from wildfire smoke with cardiovascular effects is weak. In one study, only the risk of asthma-related hospitalization increased during smoke exposure periods [63].

In contrast to long-term exposure to household air pollution (HAP) secondary to woodsmoke, the current evidence shows no association between acute exposure to smoke pollutants from wildfires and new cases of COPD or the development of biomass-induced COPD.

### 3.3. Outdoor Air Pollution Due to Other Types of Environmental Contaminants

High levels of urban air pollution increase the risk of respiratory mortality, and several studies have reported that high levels of PM of outdoor air pollution derived from traffic (PM_2.5_ and PM_10_) are associated with COPD exacerbations and prevalence. The type and concentration of the volatile chemical, biological material, and PM components of ambient air pollutants vary greatly depending on the primary source of those pollutants; therefore, more studies are needed. COPD from urban air pollution exposure likely shares some common pathogenic paths with that due to cigarette smoking and to exposure to biomass smoke; however, there is no conclusive evidence yet.

Larger roads and higher PM_2.5_ concentrations near where subjects lived were associated with greater risk for the development of COPD [64]. Long-term exposure to low-level traffic-related air pollution, especially NO_2_ and black carbon, is associated with the development of COPD [65]. COPD incidence was associated with the 35-year mean NO_2_ level in a cohort of participants living in residential areas near traffic, with stronger associations in subjects with diabetes and asthma [66], and living close to busy roads was also associated with COPD and lower lung function (FEV_1_ and FVC) in adult women. [67]

Current in vitro studies show that ambient air-derived particulate matter-induced human alveolar macrophages and airway epithelial cells produce proinflammatory cytokines and oxidative response [68,69,70]. This evidence pointed to inflammatory–oxidative mechanisms of lung damage, which involve Toll-like receptors (TLRs) 2 and 4 [71,72,73]. In addition, ambient PM_2.5_ triggers increased oxidized phospholipids in the lungs and a systemic cellular inflammatory response through TLR4/NADPH oxidase-dependent mechanisms in a mouse model [74].

## 4. The Effect of Chronic Exposure to Woodsmoke on Lung Function and Respiratory Symptoms

Various cross-sectional studies have reported that chronic exposure to household biomass smoke is associated with an increased prevalence of respiratory symptoms, reduced lung function, and the development of COPD. Ventilatory function (forced expiratory volume in 1 s, FEV_1_; forced vital capacity, FVC; and FEV_1_/FVC ratio) was significantly reduced in women cooking with biomass fuels compared to those that use LPG [75,76,77], and in male and female participants across all age-groups [78]. Chronic exposure to biomass smoke is associated with increased respiratory symptoms such as cough, wheezing, mucus overproduction, dyspnea, and chronic bronchitis [75,76,77,79]. A recent large study showed that indoor cooking with solid fuel is associated with reduced peak expiratory flow (PEF) in middle-aged and older adults from a large cohort in China over a 4-year follow-up [80]. A case–control study of asthma nested within a US rural cohort showed that frequent exposure to residential wood burning was associated with increased fractional exhaled nitric oxide (FeNO) among all individuals and with lower pulmonary function among individuals with asthma [81].

## 5. Biomass-Related COPD

### Natural History and Phenotype of Biomass-Related COPD

In contrast to smoking-related COPD, biomass-related COPD’s natural history and phenotype are poorly characterized. Ramírez-Venegas et al. [82] reported a 15-year follow-up of 112 COPD patients chronically exposed to wood-derived biomass smoke during cooking (87% female, 243 ± 122 hour-years of exposure), and 302 smoking COPD patients, 26% female, with cumulative smoking of 57 ± 32 pack-years. They observed a slower decline in lung function, absence of rapid decliners, and less variability in the lung function decline in biomass-related COPD compared with smoking-related COPD. Salvi et al. [17] confirmed the slower rate of decline in lung function among nonsmoking biomass-related COPD patients compared to smoking-related COPD patients in their two-year longitudinal study.

Several cross-sectional studies have shown that biomass-related COPD is associated with less emphysema (according to CT scans or a substantially reduced DLCO) [16,44,83,84]; a higher rate of bronchial hyperresponsiveness and response to bronchodilators [17]; more fibrosis in the lung parenchyma and in the walls of the bronchi, bronchioles, and blood vessels; and stronger anthracosis compared to smoking-related COPD [45]. Biomass-smoke exposure was typically >100 hour-years or a lifetime exposure of 10 years or more. Mahesh et al. reported a minimum threshold biomass-smoke exposure of 60 hour-years to confer a significant risk of developing chronic bronchitis in women [85]. In summary, biomass-related COPD is predominantly a small-airway disease phenotype exhibiting less emphysema, preserved lung diffusion capacity, more scarring, greater anthracosis, and a slower decline in lung function. It can be considered another phenotype of COPD.

## 6. Mechanisms of Lung Damage Due to Biomass-Smoke Exposure

Exposure to wood biomass smoke from cooking or heating typically is lifelong, starting before birth. Evidence from clinical studies on exposed disease-free individuals and in vivo and in vitro experimental models have shown that biomass smoke induces airway inflammation, systemic inflammation, increased oxidative stress, and cellular DNA damage. The following sections focus on studies investigating the effect of biomass smoke from wood combustion, the most common source of indoor biomass smoke and the leading risk factor for COPD, including studies in human patients and volunteers (Section 6.1), exposed animal models dealing with mechanisms of lung pathogenesis, and in vitro models testing potential components of specific mechanisms of pathogenesis (Section 6.2).

### 6.1. Effect of Woodsmoke Exposure on Healthy Adult Individuals

#### 6.1.1. Chronic Exposure to Woodsmoke in Healthy Adult Individuals

Overall, studies have consistently shown that chronic exposure to woodsmoke induces airway inflammation, systemic inflammation, oxidative stress, and genotoxicity, and affects populations of immune cells such as B lymphocytes in healthy individuals.

Falfán-Valencia et al. [86] examined 27 cytokines in the sera of 30 never-smokers with chronic biomass exposure (CS−/BS+), 40 smokers with no exposure to biomass (CS+/BS-), and 30 never-smokers/unexposed to biomass controls (CS-/BS-). Compared to the unexposed controls, CS-/BS+ showed higher levels of IL-1ra, IL-6, IL-8, eotaxin, IP-10, RANTES, and VEGF, which may indicate an inflammatory profile favoring an eosinophil-derived inflammatory response, whereas CS+/BS- had higher levels of IL-2, IL-9, MCP-1, MIP-1β, and VEGF. The cytokines altered in CS-/BS+ have been associated with asthma, COPD, lung fibrosis, and lung cancer. In this study, the participants self-reported their biomass exposure times by answering a standardized questionnaire.

Several cohorts of women from rural areas of West Bengal who had cooked daily with wood, cow dung cake, and agricultural waste for at least five years were compared to women who had cooked with LPG. [87,88,89,90,91,92]. Biomass users were exposed to higher indoor concentrations of PM_10_ and PM_2.5_ than LPG users, and presented increased DNA damage, increased generation of reactive oxygen species (ROS), and diminished levels of superoxide dismutase (SOD) in the buccal epithelial cells [87] and sputum cells [88]. In addition, biomass users showed an increase in the number of neutrophils, eosinophils, lymphocytes, and alveolar macrophages in the sputum [89,90,91]. In a large cohort of 635 biomass-exposed women and 452 age-matched control women, biomass users showed increased serum levels of IL-6, IL-8, TNF-α, CRP, and ROS generation, while SOD was depleted in leukocytes [92], indicating systemic inflammatory responses and increased oxidative stress due to biomass-smoke exposure. The increased systemic levels of IL-6 and IL-8 were consistent with what Falfán-Valencia et al. reported [86].

In 200 healthy nonsmoking women in rural Bangladesh exposed to household biomass smoke (assessed by measuring PM_2.5_, black carbon (BC), and carbon monoxide (CO) with personal monitors), Raqib et al. [93] found a reduction in CD19^+^ B lymphocytes and memory B cells (CD19^+^CD27^+^), with increased plasma IgE levels and reduced production of CCL5 and TNF-α by monocyte-derived macrophages. A decreased number of CD19^+^ B lymphocytes in biomass-exposed women was previously reported by Dutta et al. [94] in 2012.

Table 2 summarizes the effects of chronic exposure to woodsmoke on healthy adults.

#### 6.1.2. Controlled Short-Term Exposure to Woodsmoke in Healthy Adult Individuals

Short-term exposure to woodsmoke for periods varying between 2 and 4 h in length, with or without intervening no-exposure intervals, and with PM concentrations of 200–500 μg/m^3^, has been studied in small groups of 10–40 individuals at rest, and some of the studies added exercise intervals aiming to investigate airways and systemic inflammation, oxidative stress, DNA damage, and changes in cardiovascular physiology [34,95,96,97,98,99,100,101,102,103,104,105,106,107]. Overall, the findings have been inconsistent, with some finding airway inflammation [34,95,96,97], but not others [98,99]. Blood neutrophils, serum inflammatory cytokines, and adhesion receptors have been reported as undergoing no change after short exposure [99,100,101], while others have shown changes indicative of systemic inflammation, such as an increased number of neutrophils in the blood [34] and an increased concentration of serum Clara cell protein (CC16) [96,102]. Evidence of oxidative stress has also been inconsistent, while evidence suggests no genotoxic effect of short-term exposure to woodsmoke [101,103,104]. On the other hand, some studies suggested that short-term exposure to woodsmoke may affect cardiovascular health by causing an increase in systolic pressure, central arterial stiffness, a simultaneous reduction in heart rate variability, and an increased concentration of serum amyloid A [105,106,107].

Inconsistencies may be due to the heterogeneity in the selected endpoints, the variety of combustion conditions, the time and dose of exposure, the inclusion of exercise, and varying intervals between exposures.

### 6.2. In Vitro and In Vivo Studies on the Effect of Woodsmoke Exposure

In vitro studies have consistently shown that wood biomass smoke induces an inflammatory response, oxidative stress, cytotoxicity, genotoxicity, mitochondrial dysfunction, mucin expression, decreased epithelial barrier function, lung parenchymal damage, and endoplasmic reticulum stress.

#### 6.2.1. Biological Effects of Woodsmoke on the Airway Epithelium

Airway epithelial cells are directly exposed to pollutant components following the inhalation of biomass smoke, triggering the production of reactive oxygen species and inflammatory mediators followed by disruption of the epithelial barrier and an innate immune response. Due to the heterogeneity and diverse biological effects, particle components from other sources, such as traffic or ambient-derived particles, are not included.

Human airway epithelial cells, 16HBE, produce higher levels of proinflammatory IL1-β, TNF-α, IL-8, and IL-6 mRNA after in vitro exposure to PM_2.5_ (40 mg/L) from wood [108]. In addition, PM_2.5_-exposed 16HBE cells showed mitochondrial dysfunction and altered mitochondrial metabolism by a decreased mitochondrial membrane potential, increased expression of the cleavage protein phospho-DRP1, increased mitochondrial ROS (mtROS), decreased ATP levels, decreased oxygen consumption and glycolysis and a change in mitochondrial morphology. In vivo experiments with mice exposed to woodsmoke PM_2.5_ for six months showed an increased number of swollen mitochondria with depletion of cristae in lung tissue, confirming the mitochondrial dysfunction.

Ex vivo-cultured human lung parenchymal tissue (mostly from ex-smokers without reported disease) responded to woodsmoke PM extracts (collected near stoves during cooking, 276.1 mg/m^3^, PM_2.5_) by producing IL1-β, IL-6, IL-8, TNF-α, CCL2, CCL3, and CCL13, confirming the inflammatory effects of woodsmoke on tissue level [109]

Woodsmoke infusion (from ignited birchwood toothpicks) reduced the epithelial barrier function and E-cadherin expression, induced a loss of the cobblestone patterning, and caused a reduction in migration without affecting the viability of adenocarcinoma-derived A549 epithelial cells [110] through a p44/42 MAPK-dependent pathway. Therefore, lung epithelial cell lines or primary lung epithelial cells from noncancerous donors should also be tested.

Woodsmoke at a concentration 25-fold lower than that of CS increased the expression of mucin 5AC oligomeric mucus/gel-forming (MUC5AC) and SAM tip domain containing the ETS transcription factor (SPDEF) mRNA in mice and human airway epithelial cells (AECs) [111], especially in AECs homozygous for the p53Arg.

The results support the idea that woodsmoke increased airway mucin production more than CS and that p53 may be involved in the induction mechanism.

Water-soluble filtrates (2 mg/mL) of wood tar on mice and on human BEAS-2B virus-immortalized epithelial cells derived from normal lung tissue [112] showed increased gene expression of IL-1β, TNF-α, and IL-8; increased cell death; increased production of superoxide anions; lower rates of oxygen consumption; and a reduction in the extracellular acidification rate. In addition, mice exposed to aerosols of water-soluble fractions (2 or 10 mg/mL for 15 min) displayed increased expression of IL-1β, TNF-α, and IL-6 in lung tissue; increased levels of neutrophils, macrophages, and monocytes in the BALF; and increases in oxidative stress markers such as heme oxygenase-1 (HO-1), metallothionein-2 (MT-2), and cytochrome P450 2E (CYP2E). In addition, water-soluble wood tar fractions (containing phenolic compounds) increased total reactive oxygen species and H_2_O_2_ production, decreased the mitochondrial membrane potential (MMP), and induced oxidative damage and cell death. Whereas the wood tar organic-soluble fraction (containing more polycyclic aromatic hydrocarbons (PAHs) and oxygenated PAHs) increased superoxide anion production and decreased MMP on BEAS-2B and A549 cells [113]. Exposure to both fractions caused cell-cycle alterations in the G2/M phase induced by the upregulation of p21 and p16 and cell death.

Pine-woodsmoke particles induce cytotoxicity [114,115,116] and endoplasmic reticulum stress (ERS) [116] in human bronchial epithelial cells (HBECs), primary human lobar bronchial epithelial cells (lobar HBECs), BEAS-2B, and cancer-derived A549 cells.

Early studies showed that woodsmoke PM induced DNA damage and oxidative stress [117,118] on A549 cells, with increased levels of IL-8 [117] or without affecting IL-8 and IL-6 expression levels [118].

**Figure 1:**
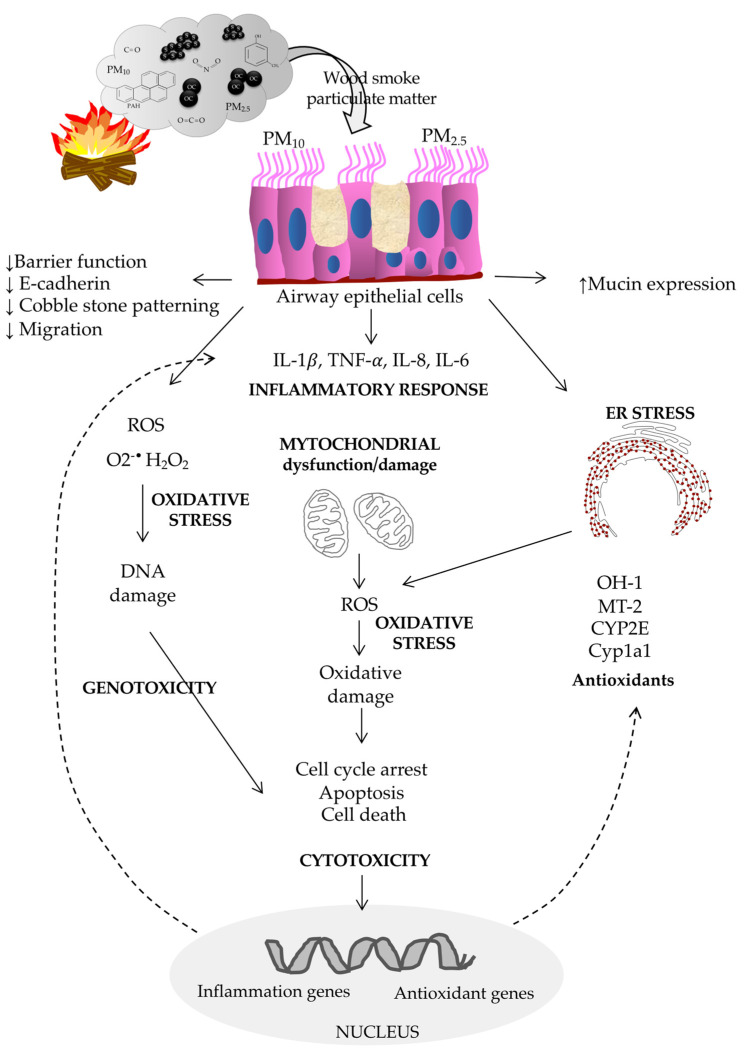
Scheme of the biological responses of human airway epithelium to woodsmoke exposure.

Table 3 and Figure 1 summarize the biological responses of human lung epithelium to woodsmoke exposure.

#### 6.2.2. Biological Effects of Woodsmoke on Alveolar Macrophages (AMs)

The function of alveolar macrophages is to detect, phagocytose, and eliminate all foreign material from the airway space, which includes PM_2.5_ and PM_10_ from inhaled air pollution [119,120,121,122]. AMs can phagocytose unopsonized environmental particles through scavenger receptors, mainly the class A scavenger receptor (SR-A) and the macrophage receptor with collagenous structure (MARCO) [123,124]. AMs also phagocytose environmental particles containing bacteria or microbial material, such as endotoxins, through Toll-like receptors (TLRs), mainly TLR4 and TLR2 [73]. After ambient PM exposure, AMs are activated and, together with airway epithelial cells, initiate an inflammatory response that involves the production of reactive oxygen species and inflammatory mediators. However, alveolar macrophages produce higher levels of inflammatory mediators such as IL-1, IL-6, and TNF-α than airway epithelial cells when exposed to the same dose of PM, suggesting that alveolar macrophages are the predominant inductors of the inflammatory response [125]. Atmospheric particles induce proinflammatory cytokines (such as IL-1β, IL-6, IL-8, TNF-α, and GM-CSF), chemokines macrophage (such as inflammatory protein 2 MIP-2, and regulated upon activation, normal T-cell expressed and secreted, RANTES), and interferons [119,126]. These cytokines promote the infiltration of neutrophils, dendritic cells, and T cells while inducing the recruitment and maturation of monocytes from the bone marrow [68,127]. However, the cytokines produced by PM-exposed AM depend on the PM’s size and chemical composition, source, time of exposure, and perhaps the macrophage phenotype [126,128]. Upon activation, macrophages acquire polarized M1 or M2 phenotypes that induce Th1- or Th2-mediated immune responses, respectively. Exposure to environmental PM_2.5_ induces M1 polarization in bronchoalveolar lavage fluid (BALF) from PM-exposed mice [129]. However, in a model of COPD murine model, environmental PM_2.5_ promotes M2 polarization in AMs [130].

All these studies have addressed the effect of PM derived from diverse sources of air pollution (e.g., ambient particulate matter, urban particulate matter, diesel exhaust particles) on alveolar macrophages [69,70,121,122,125,126,131].

Similar to AMs exposed to atmospheric particulate matter [121], AMs and monocyte-derived macrophages (MDMs) from volunteers exposed to household air pollution can phagocytize woodsmoke particles dose-dependently [132]. In addition, woodsmoke particles induce a dose-dependent production of IL-6 and IL-8 in both cells, while reducing the phagocytosis capacity and oxidative burst activity in AMs [132].

MDMs from BS-COPD and CS-COPD patients exhibit defective phagocytosis [133] with higher bacterial loads of *S. pneumoniae*, *H. influenzae*, and *P. aeruginosa* in induced sputum and with a decline in spirometric lung function indices compared to healthy subjects.

Infiltrated AMs in the BALF of rats chronically exposed to woodsmoke-derived PM_1_, PM_2.5_, and PM_10_, [134] produced increased levels of IL-1β, TNFα, and LIX after one month of exposure, with only increased levels of VEGF after six months of exposure. After exposure, AMs also showed a decreased expression of the M2 marker CD206 after four days, a recovery in CD206 expression after one month, and an increase in expression levels after six months of exposure. The increased expression of CD206 was accompanied by increased TGF-β1 and p-Smad3 expression, leading the authors to suggest that chronic biomass PM affects alveolar macrophage polarization and activation. Bazzan et al. [135] found a dual polarization of human alveolar macrophages that progressively increases with smoking and COPD severity in CS-COPD patients. In contrast, Shaykhiev et al. [136] reported that AM of healthy smokers exhibited M2 polarization.

#### 6.2.3. Biological Effects of Woodsmoke on Fibroblasts and Endothelial Cells

Due to its intrinsic toxicity, woodsmoke affects other cell lineages in the airways, such as fibroblasts and endothelial cells. Its effects include cell activation, the expression of inflammatory cytokines, the deposition of extracellular matrix components, and genotoxicity.

Woodsmoke PM (0.3–10 mm in size) enhanced proinflammatory IL-8 production, increased pERK1/2 levels, and led to higher deposition of the extracellular matrix components perlecan and fibronectin in human lung fibroblasts (from donors with thoracic malignancies or undergoing lung transplantation) [137].

Woodsmoke particles (PM size 113 nm, high PAH content) induced the adhesion of monocytic THP-1 cells onto human umbilical endothelial cells (HUVECs) in co-cultures, which correlated with increased expression of VCAM-1 on exposed HUVECs and increased expression of the TNF and IL8 mRNAs in THP-1 cells. [138] In addition, woodsmoke increased DNA strand breaks and oxidized guanines, with low cytotoxicity toward HUVECs. This study showed that woodsmoke induces oxidative damage to endothelial cell DNA and the adhesion of monocytes, with the activation of both cell types.

#### 6.2.4. Potential Role of Transient Receptor Potential (TRP) in the Cytotoxicity and Endoplasmic Reticulum Stress (ERS) Response Induced by Woodsmoke Particles in Human Lung Epithelial Cells

Woodsmoke particles generated from pine wood are highly cytotoxic to human bronchial epithelial cells in vitro, induce ERS and activate transient receptor potential ankyrin-1 (TRPA1) and transient receptor potential vanilloid-3 (TRPV3) ion channel receptors differentially in human bronchial epithelial cells [114,116,139,140]; however, evidence on their role in cytotoxicity and ERS has been conflicting.

Pine- and mesquite-woodsmoke particles activate TRPV3 in several human normal bronchial epithelial cells and A549 cells, with more cytotoxicity in BEAS-2B cells overexpressing TRPV3 (TRPV3-OE BEAS-2B), and partial protection by addition of antagonists of TRPV3 [114]. Using antagonists for TRPA1, TRPAV1, and TRPV4 does not affect woodsmoke particle-induced cytotoxicity.

Further, pine-woodsmoke particles caused cytotoxicity, activation of TRPA1 and TRPV3, and endoplasmic reticulum stress (ERS) responses in lobal human lobar bronchial epithelial cells (HBECs) and BEAS-2B [116]. TRPA1 transcripts remained low after treatment with woodsmoke particles, while TRPV3 transcripts increased, indicating distinct transient expression. Contrary to the previous findings, the inhibition of TRPV3 increased woodsmoke particle-induced cytotoxicity in lobal HBECs, while the inhibition of TRP1 decreased it. In addition, TRPV3 antagonists increased ERS in lobal HBECs, while TRP1 antagonists decreased it. Furthermore, the overexpression of TRPV3 in TRPV3-OE BEAS-2B cells conferred resistance to ERS by woodsmoke particles, whereas TRPV3 knockdown in BEAS-2B sensitized the cells to the induction of ERS [116]. These results indicate that TRPA1 promotes cytotoxicity and ERS response induced by woodsmoke particles, and TRPV3 protects against these phenomena, which are inconsistent with previous findings.

#### 6.2.5. TRPA1 and the Epidermal Growth Factor Receptor (EGFR) Signaling Pathway Are Involved in MUC5AC Expression and Cytotoxicity Induced by Woodsmoke Particles in Human Bronchial Epithelial Cells

Woodsmoke particles stimulated MUC5AC overexpression by human bronchial epithelial cells [111,115,141]. However, mechanistically, these studies found that different components of the EGFR pathway were involved in woodsmoke-particle-induced MUC5AC.

Memon et al. [115] found that pine-woodsmoke particles (PM_2.5_) and coniferaldehyde, a highly concentrated component and TRPA1 agonist, induced cytotoxicity and the expression and secretion of MUC5AC by primary human bronchial epithelial cells (HBECs). The synthetic TRPA1 antagonist A967079 attenuated the overexpression of MUC5AC after treatment with pine-woodsmoke particles, indicating that TRPA1 is involved but not essential.

Pine-woodsmoke particles and coniferaldehyde increased the expression of the EGFR signaling components epigen (EPGN), heparin-binding epidermal growth factor (HB-EGF), and phosphor-EGFR, and induced the accumulation and perinuclear/nuclear localization of β-catenin. Furthermore, antagonists for EGFR/ErbB1, p38 MAPK, and GSK3β inhibited but did not abolish the MUC5AC overexpression induced by woodsmoke particles. These results suggest that the EGFR-dependent activation of p38 MAPK, GSK3β, and β-catenin contributes to pine-woodsmoke-particle-induced MUC5AC overproduction in HBECs, as well as the activation of TRP1. The authors suggested that MUC5AC production results from the selective and coordinated activation of TRPA1 and EGFR. A potential coordinated effect between TRP1 and EGFR activation could be investigated by inhibiting one participant (e.g., by using an effective antagonist, siRNA, or knockdown) while testing the participation of the other in the induction of MUC5AC overexpression by woodsmoke particles.

Woodsmoke PM_2.5_ also induced MUC5AC expression in human primary epithelial cells (carcinoma mucoepidermoid-derived NCI-H292 cell line) and in the airways of rats exposed to woodsmoke for three months. Expression of MUC5AC was inhibited using an EGFR-selective tyrosine kinase inhibitor, an EGFR-neutralizing antibody, the ERK kinase inhibitor PD98059, and specific neutralizing antibodies for AREG [141]. In contrast to the study by Memon et al. [115], woodsmoke particles increased the AREG mRNA and protein levels in epithelial cells. This discrepancy may be related to the use of carcinoma-derived cells instead of normal epithelial cells in this study. This study showed that woodsmoke PM_2.5_ induced MUC5AC via the AREG–EGFR–ERK pathway in the cancer-derived NCI-H292 cell line.

Overall, both studies showed that the activation of the EGFR signaling pathway contributes to woodsmoke-particle-induced MUC5AC expression and cytotoxicity in human bronchial epithelial cells, in addition to the contribution of TRPA1, which may or may not be coordinated with it.

#### 6.2.6. Potential Role of Toll-Like Receptors (TLRs) in Inflammation Induced by Woodsmoke Particles

Mice exposed to woodsmoke-derived particles for 24 h (acute exposure, by nose aspiration during anesthesia) showed increased proinflammatory cytokine production, neutrophilic inflammation, and hyperresponsiveness. Meanwhile, in treated mice, subchronic exposure (three doses per week and examination eight weeks after exposure) induced eosinophilic inflammation and alveolar destruction. Only subchronic wood-particle exposure resulted in significant increases in airspace enlargement in the lungs of the treated mice. Furthermore, subchronic exposure to woodsmoke particles increased eosinophil cytokines (IL-5 and eotaxin). In contrast, exposure to cow-dung particles increased neutrophil and macrophage chemokines (G-CSF, MIP-1a, MIP-1b, IP-10, and IFNγ), indicating a differential effect on mice according to the fuel source [130]. Using only the acute exposure model, TLR2-, TLR3-, TLR4-, TLR5-, and IL-1R–deficient mice were exposed to wood- or dung-smoke particles, and the measurement of neutrophils in the BALF indicated that IL-1R, TLR4, and TLR2 are necessary to elicit that inflammatory response in mice [142].

It would be interesting to know the role of TLRs in the inflammatory responses induced by chronic exposure to woodsmoke and obtain models exposing awake animals in chambers controlling concentrations of particulate matter and other toxics. Chronic exposure to woodsmoke associated with developing COPD-like lesions in animal models [134].

## 7. Mechanisms of Pathogenesis Involved in Biomass-Related COPD

**Figure 2:**
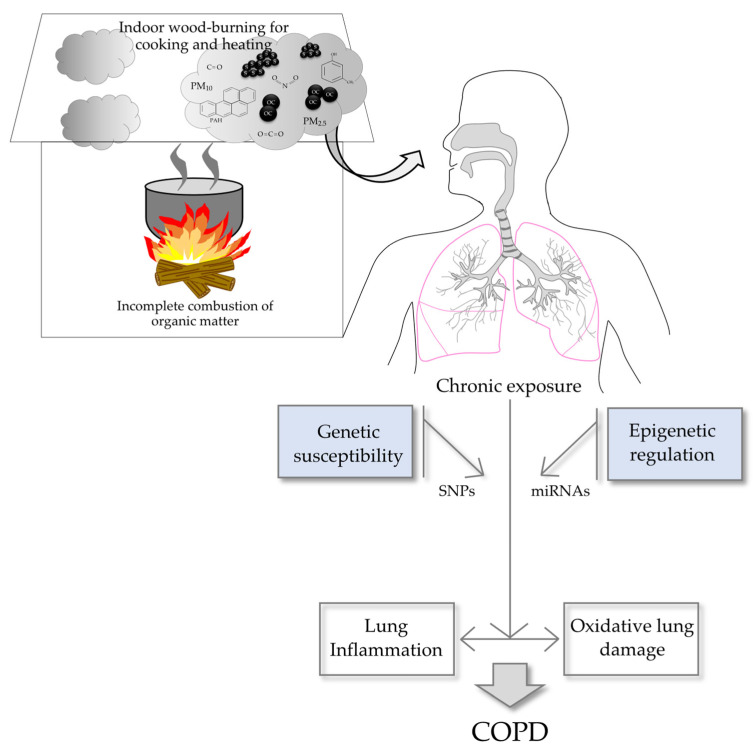
Biomass-related COPD is a complex and multifactorial disease that develops after the combination of chronic exposure to the hazardous components of woodsmoke and genetic and epigenetic factors of susceptibility. Organic carbon (OC), soot (s).

In addition to smoking, chronic exposure to woodsmoke is the most well-known environmental burden contributing to COPD development. However, COPD is a complex and multifactorial disease that develops after the inhalation of toxics, most often tobacco smoke or biomass smoke, and has genetic and epigenetic factors of susceptibility (Figure 2). Studies on nonsmoking COPD patients with chronic exposure to woodsmoke have shown changes in the inflammatory response, increased DNA damage, oxidative stress, and genetic and epigenetic susceptibility factors.

### 7.1. Inflammation

In contrast to those for COPD related to cigarette smoke, few studies have consistently assessed the inflammatory profile and potential inflammatory mechanism of pathogenesis in COPD related to wood-biomass-smoke exposure.

Shyam et al. [143] examined the levels of the proinflammatory cytokines IL-1β and TNF-α and the potential correlation with the severity of airflow limitation in 40 patients with COPD associated with cigarette smoke (CS-COPD) and 40 biomass-smoke exposure (BS-COPD) compared to 80 healthy controls. COPD patients presented higher levels of IL-1β and TNF-α compared to controls. Among the COPD cases, there was a negative linear relationship between TNF-α and IL-1β levels with the FEV_1_ and 6 min walking distance. According to linear regression, TNF-α levels were associated with CS-COPD, while IL-1β levels were associated with both CS- and BS-COPD. In addition, BS-COPD patients had higher levels of IL1-β, but lower levels of TNF-α than the CS-COPD, suggesting differences in the inflammatory responses between them.

In a comparison of 29 BS-COPD female subjects, 24 BS-exposed women without COPD (BS-CTRL), 23 CS-COPD male patients, and 22 male smokers without COPD (CS-CTRL), the chemokines CCL15, CCL27, and CXCL13 were significantly lower in the BS-COPD (vs BS-CTRL) whereas the chemokines CCL1, CCL7, CCL15, CCL17, CCL19, CXCL2, IFNγ, and MIF were significantly different between the CS-COPD and CS-CONTROL [144].

Sputum eosinophilia (sputum eosinophils ≥ 3%) in 28 BS-COPD cases, was more common than in 85 CS-COPD while both BS-COPD and CS-COPD presented neutrophils in sputum [145].

In a comparison of 31 BS-COPD, 49 CS-COPD, 46 COPD patients with both exposures, and 52 healthy controls [146], COPD with combined CS + BS exposure showed lower oxygenation spirometric function and DLCO. BS-COPD and combined exposure-COPD patients showed higher levels of IgE, suggesting a Th2-type response, whereas smokers with COPD showed higher levels of CRP and fibrinogen than BS-COPD patients [146].

BS-COPD and CS-COPD have higher inflammatory responses compared to healthy controls, but the inflammatory profile showed some differences between the subgroups of COPD patients [147]. The BS-COPD group expressed significantly lower levels of IL-6, IL-8, and IL-5 than CS-COPD.

Serum levels of secretoglobin family 1A member 1 (SCGB1A1) also known as club cell protein 16 or CC16, were lower in both the BS-COPD and CS-COPD compared to the CS controls and healthy controls. In addition, the SCGB1A1 levels were positively correlated with FEV1, FVC, and exercise capacity [148].

Plasma levels of matrix metalloproteinase-(MMP)-1, MMP-7, MMP-9, MMP-9/TIMP-1, and CRP were similar in women with BS-COPD and CS-COPD but higher than those in unexposed controls [149].

Table 4 summarizes the studies on inflammatory markers in biomass-related COPD. Although the results depict inflammatory responses, those are difficult to compare because different studies primarily examined different components (Table 4). Further, the results were inconsistent between studies when similar endpoints were tested. For example, sputum eosinophilia and higher IgE levels in BS-COPD compared with CS-COPD have been reported in some studies, but not in others (Table 4). The same inconsistency is observed with high levels of IL-1β and TNF-α in both BS-COPD and CS-COPD, and elevated IL-6 and IL-8 in BS-COPD and CS-COPD (Table 4). Further studies on the inflammatory response in BS-COPD are needed, as inflammatory features may be distinct from this COPD phenotype.

### 7.2. Oxidative Stress and DNA Damage

Patients with BS-COPD (*n* = 30) and CS-COPD (*n* = 30, ex-smokers) had higher plasma levels of malondialdehyde (MDA) and increased activity of superoxide dismutase (SOD) compared to unexposed healthy controls (*n* = 30), with no significant differences in glutathione peroxidase (GPx), glutathione reductase (GR), and glutathione-S-transferase (GST), indicating a similar pattern of oxidative stress [150]. In addition, MDA levels and SOD activity were inversely correlated with FEV1 in both groups of COPD patients.

Higher levels of DNA strand breaks in peripheral blood mononuclear cells and higher levels of plasma MDA and protein carbonyl (PC) have been found in both BS-COPD patients and CS-COPD patients compared to controls. DNA damage levels were higher in CS-COPD compared to BS-COPD [151].

### 7.3. MicroRNAs

#### 7.3.1. MicroRNAs in COPD

The development of COPD involves complex interactions between environmental insults and genetic/epigenetic determinants. Among the epigenetic regulatory mechanisms, microRNAs (miRNAs) have been extensively studied as master gene regulators in health and disease. miRNAs are small noncoding RNA molecules (22 nucleotides long) that regulate the expression of genes involved in multiple biological processes at the posttranscriptional level by blocking the translation of target messenger RNAs (mRNAs).

Several studies examining changes in the microRNA profiles of bronchial biopsies, lung tissue, airway cells, sputum, and peripheral blood in COPD patients have linked these changes to disease status, diagnosis, survival, pathogenesis, and response to corticosteroid treatment [152,153,154,155,156,157]. Furthermore, miRNA and mRNA expression studies on COPD patients have revealed miRNA–mRNA network interactions associated with potential pathogenesis mechanisms [153,158]. Therefore, increasing evidence suggests that miRNAs may play a crucial role in the pathogenesis and development of COPD. Notably, these studies included ex-smoking or smoking COPD patients, with only one study including nonsmoking COPD patients with no occupational exposure to dust, soot, or coal dust [158].

#### 7.3.2. MicroRNAs in Biomass Smoke-Related COPD

In contrast to cigarette smoke-related COPD, few studies have aimed to specifically elucidate the role of miRNAs in biomass-related COPD (Table 5).

The expression of miR-22 in the sera of 25 BS-COPD female patients was decreased compared to those in 25 with CS-COPD [159]. Accordingly, the BS-COPD group had significantly elevated levels of histone deacetylase 4 (HDAC4), a predicted target of miR-22, compared with the CS-COPD. Levels of HDAC4 were positively correlated with carbon monoxide diffusing capacity as a percentage of predicted (DLCOsb%) in both groups of COPD patients. However, HDAC4 targeting by miR-22 or disease-free controls were not studied.

In comparing 96 miRNAs in three serum samples from each of the three study groups (BS-COPD, CS-COPD, and healthy controls) [160], six differentially expressed miRNAs were subsequently tested by qPCR using 25 serum samples from each study group. Out of four validated miRNAs, the authors selected miR-34a-5p, downregulated in BS-COPD compared to CS-COPD, for further analysis, and chose Notch1 (a predictive target of miR-34a-5p) for quantification in the sera of the same cohort by ELISA. They found increased levels of Notch1 in BS-COPD compared with CS-COPD.

Based on those results, the authors inferred that the downregulation of miR-34a-5p induces upregulation of Notch1, which may play a role in the differentiation of the airway epithelium associated with bronchitis in BS-COPD.

However, the downregulation of miR-34a-5p among COPD patients with different risk factors does not imply an association with the disease status. Furthermore, the downregulation of miR-34a-5p cannot be associated with the risk factor either, as differential expression was not detected between disease-free individuals with different exposure types. Notch1 targeting by miR-34a-5p was not assessed but was based on computational target prediction and (possibly) other studies, such as that of Long et al. [161]. Rather, miR-34 may be useful for discriminating between COPD patients with different risk factors, but also for supporting the notion that biomass-related COPD may have a different phenotype and should be further investigated. On the other hand, miR-374a-5p was downregulated in BS-COPD compared to unexposed controls, while miR-191-5p was upregulated in BS-COPD patients compared to controls exposed to biomass. Further examination of these miRNAs may shed light on whether circulating miRNAs are involved in the diagnosis, phenotype, or pathology of BS-COPD patients. However, these findings were not discussed in this study.

Díaz-Peña et al. [162] analyzed 2069 miRNAs in the sera of 15 BS-COPD and 15 CS-COPD patients using high-throughput sequencing without quantitative validation. Disease-free controls were not included. They reported 45 miRNAs differentially expressed in BS-COPD compared to CS-COPD. Then they performed a gene ontology (GO) analysis to find related biological mechanisms. Finding a different miRNA expression profile in BS-COPD compared to CS-COPD is hypothesis-generating, but causality would be supported further by comparison with disease-free controls, quantitative verification of sequencing data, and larger and independent validation cohorts.

Together, these three studies provide preliminary evidence that BS-COPD patients might have a different miRNA expression profile from CS-COPD patients and possibly a different phenotype.

Nevertheless, further studies are needed to identify and validate miRNAs potentially associated with disease status. Once miRNAs are discovered and validated, mechanistic studies will assess the biological role in the pathogenesis of biomass-related COPD.

### 7.4. Genetic Susceptibility

#### 7.4.1. COPD Genetic Studies

There is strong evidence for the association of multiple genes with lung function and susceptibility to COPD. Early family-based studies showed COPD clusters in families in response to smoking, with a threefold increased risk of airway obstruction in smoking first-degree relatives of patients with severe COPD, but not nonsmokers [163,164]. Twin [165] and unrelated population-based studies [166] indicated significant heritability for COPD in smokers.

Severe alpha-1 antitrypsin (A1AT) deficiency, a rare genetic variant, is the best-described genetic risk factor for COPD and accounts for approximately 1% of COPD cases, especially in individuals of European origin [167].

Multiple genomic regions associated with COPD susceptibility have been identified by genome-wide association studies (GWASs), including single-nucleotide polymorphism (SNP) genotyping [168]. Multicenter longitudinal studies, such as Genetic Epidemiology of COPD (COPDGene), Subpopulations and Intermediate Outcome Measures in COPD Study (SPIROMICS), Evaluation of COPD Longitudinally to Identify Predictive Surrogate Endpoints (ECLIPSE), and subsequent joint meta-analyses have enabled the validation of more than 20 loci associated with COPD status [168,169,170,171,172,173]. Among the loci that have been replicated by different studies are HHIP (4q31), FAM13A (4q11), and CHRNA3/CHRNA5/IREB2 (15q25) [168]. Notably, these studies included COPD patients with a history of smoking, but not COPD patients who were nonsmokers.

Wain et al. [174] published the first GWAS that included COPD cases from heavy and never-smokers from subjects of European descent from the UK Biobank Lung Exome Variant Evaluation (UK BiLEVE) study cohort. They reported six novel genome-wide significant signals of association with extremes of FEV_1_ in both that showed associations with COPD in both ever-smokers and nonsmokers. To date, four other combined GWAS meta-analyses have been published that included the British BiLEVE cohort [175,176,177,178], but no information was provided on whether exposure to biomass smoke or another pollutant occurred in the cohort of nonsmokers.

#### 7.4.2. Genetic Studies on Biomass Smoke-Related COPD

A few recent case–control studies performed by the same research group have analyzed associations of selected SNPs with COPD status in never-smoking women with long-term biomass exposure due to wood burning for indoor cooking in rural Mexico (Table 6). Women exposed to biomass smoke in rural areas of Mexico and several other Latin-American countries often have strong Amerindian ancestry.

The rs2070908 in HSP90B1 (in the heat shock protein 90 gene complex) was associated with a decreased risk (*p* < 0.01, OR = 0.6) of suffering from COPD among the subjects chronically exposed to biomass, and the rs13296 GG genotype with a lower risk (*p* = 0.01, OR = 0.22) of severe COPD in smokers [179].

The rs2275913 and rs8193036, two selected interleukin 17A (IL-17A) SNPs, were associated with an increased risk of CS-COPD (*p* < 0.01; OR = 2.91) and BS-COPD (*p* = 4.52 × 10^−17^, OR = 1.11; *p* = 3.15 × 10^−17^, OR = 1.11) [180]. IL-17A has previously been associated with chronic inflammation and the progression of COPD [181].

The rs13118928 GG genotype (*p* = 0.021; OR = 0.51; 95% CI: 0.27–0.97) and the rs13118928–rs1828591 (GG) haplotype (*p* = 0.04; OR = 0.65; 95% CI: 0.43–0.98) from the Hedgehog-interacting protein (HHIP) gene were associated with a decreased risk of COPD in women exposed to biomass smoke [182].

The HHIP loci are among the replicated loci associated with COPD in several studies [168]. rs1828591 was previously associated with FEV_1_/FVC in a general population cohort [183] and a Chinese COPD smoking cohort [184], while rs13118928 and rs1828591 were associated with FEV_1_ in a familial early-onset COPD cohort [172]. Although the rs13141641 SNP of HHIP was associated with COPD status [169,176], no significant association between rs13118928 and rs1828591 and COPD risk had previously been reported.

The alpha-1 antitrypsin (A1AT) protein polymorphisms PiS (rs17580) were associated with CS-COPD (*p* < 0.001, OR = 2.16) and BS-COPD (*p* < 0.0001, OR = 11.46). Severe deficiency of alpha-1 antitrypsin is uncommon in women exposed to biomass in Mexico, often with a strong Amerindian ancestry. Another study found a marginal association between the CT haplotype in the SERPINA1 gene rs1303-rs709932 and a reduced risk of COPD related to smoking (*p* = 0.048, OR = 0.81). Still, no significance was found for COPD related to biomass smoke [185].

The rs1800629 GA genotype for tumor necrosis factor TNF was associated with susceptibility to CS-COPD, but no significance was found for BS-COPD [186]. TNF polymorphisms have been associated with the clinical phenotype and progression of smoking-related COPD, such as rs361525 [187] and rs1800629 [188,189], but no significant association between TNF SNPs and COPD in nonsmoking patients had been previously reported. Also, elevated TNF-α levels have been linked to the inflammatory response in COPD in several studies [190].

The previous studies provide data on a possible association between selected SNPs and genetic susceptibility to COPD in a population of nonsmokers, primarily women chronically exposed to wood biomass smoke (Table 6). The authors tested few SNPs, and the size of the analyzed cohorts was limited.

### 7.5. Summary of the Pathobiology of Biomass Smoke-Induced COPD Compared to Cigarette Smoke-Induced COPD

In addition to the similarities in the hazardous particulate matter and chemical compounds between biomass and cigarette smoke, both insults require long exposure to induce COPD development. However, differences in inhalation patterns (nasal in biomass smoke and deep oral in tobacco smoke) may contribute to a less severe degree of lung tissue damage from biomass smoke than from cigarette smoke. Both biomass and cigarette smoke induce damage and injury at the cellular, tissue, organ, and systemic levels; however, not all exposed individuals develop the disease as it is clinically defined. Therefore, there are intrinsic factors contributing to the development of COPD.

In searching for differences and similarities in the pathobiology of these different phenotypes, it is relevant to compare their known characteristics. Table 7 summarizes the pathobiology of biomass smoke-related COPD compared to cigarette smoke-related COPD [192,193,194].

The information described in this review points to classic inflammatory–oxidative mechanisms of lung damage and the potential role of genetic susceptibility and miRNAs associated with chronic biomass-smoke exposure. Therefore, we extrapolated the model of inflammatory–oxidative damage from the best-known cigarette smoke-related COPD for comparison. However, there are new insights regarding the pathophysiology and molecular and cellular mechanisms of the pathogenesis of COPD in smokers. Besides the airway inflammation model of COPD pathogenesis, emerging literature indicates that airway remodeling and epithelial–mesenchymal transition (EMT) are driving mechanisms of airway fibrosis disease in COPD in smokers [195,196,197]. Moreover, studies have revealed the role of chronic colonization or recurrent infections by a specific group of bacteria in luminal inflammation and oxidative stress on the epithelium in smokers and smoking COPD patients [198,199,200]. Therefore, further research on mechanisms of lung damage and the development of COPD secondary to biomass-smoke exposure may include these potential driving mechanisms of pathology.

## 8. Potential Phenotype-Driving Therapeutics

Targeted therapies based on phenotypes are common, aiming to enhance benefits and reduce adverse consequences of treatments. COPD research is particularly challenging due to COPD’s heterogeneous nature and basically syndromic diagnosis. Most studies on COPD derive from tobacco smokers, and few identify the clinical, histopathological, pulmonary function, and medical imaging characteristics of biomass-related COPD patients, a potentially new phenotype [10,15,17].

In biomass-related COPD, the inflammatory response and other pathogenesis mechanisms have not been fully characterized, and no specific clinical trials have yet been reported for biomass-related COPD.

Scattered reports of bronchial hyperresponsiveness, eosinophilia, or increased IgE suggest that inhaled corticosteroids (ICSs) might be a reasonable strategy to test [10].

Eliminating biomass-smoke exposure, or reducing indoor pollution considerably, is important for primary and secondary prevention [201], but it is difficult to achieve as household air pollution is mostly associated with poverty, and improved biomass stoves in most places lack long-term acceptance.

Recently, Siddharthan et al. [202] published a randomized, double-blind, placebo-controlled study evaluating the clinical efficacy and cost-effectiveness of low-dose theophylline for the management of biomass-associated COPD (moderate to severe) in central Uganda. The reasoning was that the recommended inhaler-based therapy for COPD is not available or affordable in low- and middle-income countries; one option is to use low-dose theophylline—an oral, once-daily therapy. Therefore, the study was designed based on availability and affordability, not necessarily on a phenotype-driven strategy.

## 9. Discussion and Conclusions

Despite the high burden of biomass-related COPD, few studies have evaluated the molecular, genetic, and epigenetic mechanisms underlying the pathogenesis. Significant efforts have been made to identify this phenotype’s clinical, histopathological, and pulmonary imaging characteristics, but a phenotype-derived drug treatment has not been proposed to date. To date, the only recommended intervention is the elimination of biomass exposure, and prescribed treatments are extrapolations of known studies on tobacco smokers with COPD.

Importantly, biomass smoke contains similarly high concentrations of hazardous particulate matter and chemical compounds compared to cigarette smoke; however, variations in the pattern of inhalation and differences in the concentration and deposition of harmful components in the airways and lungs may contribute to the different characteristics of biomass-related COPD compared to smoking-related COPD. As for smoking-related COPD, exposure to toxic environmental risk factors alone is insufficient for developing biomass-related COPD. Instead, an inflammatory response that damages the airways and lungs and a complex combination of intrinsic factors in each individual is required. The relevance of elucidating such mechanisms may derive in more specific biomass-related COPD treatments.

In conclusion, more research is needed to elucidate the mechanisms of biomass-induced pathogenesis and susceptibility to COPD and other lung conditions. Furthermore, more data on exposure–response relationships from well-designed longitudinal studies in children, adolescents, and adults are needed to capture the adverse effects associated with exposure to biomass before birth and at an early age and the natural history of COPD development. In studies of the inflammatory response in biomass-related COPD, the assessment of homogeneous and disease-relevant biological endpoints by comparing them to those already discovered for smoking-related COPD may be of help. Genome-wide association studies on biomass-related COPD and association with Amerindian and other ethnic ancestries are needed to discover a potential gene association with lung function and susceptibility to COPD. Similar to those for smoking-related COPD, studies examining changes in microRNA profiles should be analyzed to elucidate possible associations with disease status, diagnosis, survival, and pathogenesis.

We look forward to future advances in biomass-related COPD research and clinical applications.

## Figures and Tables

**Table 1:** Health-hazardous chemicals and particulate matter present in wood smoke.

Compound	Pollutant (Species)	Health Effect
Particulate matter (PM)	Inhalable particles PM_10_	All-cause and cause-specific mortality risk, worsening of respiratory diseases, reduced lung function.
	Fine particles PM_2.5_	
	Particulate organic carbon	Toxic
Inorganic compounds	Carbon monoxide (CO)	Poison
	Sulfur dioxide	Lung damage
	Nitrogen dioxide (NO_2_)	Lung damage
	Ozone (O3)	Irritant, lung damage
	Carbon dioxide (CO_2_)	Irritant, proinflammatory, cognitive effects
Hydrocarbons, alkenes	Volatile organic compounds (C2–C7)	Cancer and others
	1,3-Butadiene	Carcinogenic
Hydrocarbons, aromatics	Benzene (monoaromatic)	Poison, toxic, irritant, carcinogenic
	Styrene (monoaromatic)	Irritant, potentially carcinogenic
	Toluene	Addictive
	Phenol	Poison, toxic
	Cresol	Toxic, corrosive
Polycyclic aromatic hydrocarbons (PAHs)	Benzo[a]pyrene	Toxic, highly carcinogenic
	Benzo[a]anthracene	Toxic, carcinogenic, mutagenic
	Dibenzo[a,h]pyrene	Toxic
	Dibenzo[a,h]anthracene	Toxic
	benzofluoranthenes	Toxic
	Chrysene	Toxic
	Pyrene	Toxic
	Fluoranthene	Toxic
	Phenanthrene	Toxic, carcinogenic
	Naphthalene	Toxic, carcinogenic
	Anthracene	Toxic
Aldehydes	Formaldehyde	Toxic, carcinogenic
	Acetaldehyde	Toxic, potentially carcinogenic
	Acrolein	Toxic
	Furfural	Irritant
	Propionaldehyde	Toxic
Other chemicals	Dioxin	Toxic, carcinogenic, teratogenic

Data adapted from [20,21,23,24,25,26].

**Table 2:** Summary of the studies analyzing the effects of chronic exposure to biomass smoke (BS) on healthy adults.

Analysis	Study Population	Size (n), Biomass Smoke vs. Controls	Findings	Reference
27 cytokines in the sera	All Female. BS: biomass exposure ≥ 100 hour-years. CS: smoking ≥ 10 pack-years. Controls: without exposure.	30 vs. 40 vs. 30	BS+ showed higher levels of IL-1ra, IL-6, IL-8, eotaxin, IP-10, RANTES, and VEGF, while CS+ had higher levels of IL-2, IL-9, MCP-1, MIP-1β, and VEGF, compared to controls.	[86]
DNA damage (comet assay) and oxidative stress (ROS and SOD) in buccal epithelial cells (BEC). Measurement of PM_10_ and PM_2.5_	BS: Women cooking daily for 2.5–5.5 h, 5 years or more with firewood, dung, or agricultural wastes. Controls: Women cooking with cleaner fuel LPG.	85 vs. 76.	Chronic inhalation of BS elicits increased oxidative stress and extensive DNA damage in BEC.	[87]
DNA damage (comet assay) and oxidative stress (ROS and SOD) in sputum cells. Measurement of PM_10_ and PM_2.5_ and benzene	BS: Women cooking daily with wood, cow dung cake and agricultural refuse for the last 5 years or more. Controls: Women cooking with cleaner fuel LPG.	56 vs. 49	Chronic inhalation of BS increased oxidative stress and DNA damage in inflammatory and epithelial cells in sputum.	[88]
Blood and sputum neutrophil number. Plasma levels of TNF-α, IL-6, IL-12, and IL-8. Myeloperoxidase activity in blood neutrophils. ROS and SOD in leukocytes. Measurement of PM_10_ and PM_2.5_	BS: Women cooking daily for 2.5–5.5 h exclusively with wood, cow dung, and agricultural refuse, for the past 5 years or more. Controls: Women cooking with cleaner fuel LPG.	142 vs. 126	Chronic inhalation of BS increased number of neutrophils in sputum and blood, levels of circulating cytokines and oxidative stress.	[89]
Immune leukocytes cells in blood	BS: Women cooking daily with unprocessed biomass such as cow dung cake, wood, dried leaves. Controls: Women cooking with cleaner fuel LPG	434 vs. 385	Chronic inhalation of BS suppressed the total number of T-helper (CD4^+^) cells and B (CD19^+^) cells and increased number of CD8+ T-cytotoxic cells, Treg cells and CD16^+^CD56^+^ natural killer (NK) cells.	[94]
Serum IL-6, CRP, TNF-α and IL-8. Arterial blood pressure	BS: Never-smoking women that cooked exclusively with solid, unprocessed biomass such as cow dung cake, wood, dried leaves, jute stick, hay, and paddy husk. Controls: Women, that cooked with cleaner fuel LPG	635 vs. 452	Chronic inhalation of BS elevated IL-6, IL-8, TNF-α, CRP, and ROS levels, while SOD was depleted by 41.5%. Greater prevalence of hypertension and tachycardia compared to their LPG-users. PM_10_ and PM _2.5_ levels associated with markers of inflammation, oxidative stress, and hypertension.	[92]
Oxidative stress (ROS and SOD); levels IL-6, IL-8, TNF-α; neutrophils, lymphocytes, eosinophils and macrophages in sputum samples. Measurement of PM_10_ and benzene exposure by measuring t,t- MA)	BS: Women cooking with biomass daily 2.5–5.5 h the past 5 years or more. Controls: Women cooking with cleaner fuel LPG.	196 vs. 149	Chronic inhalation of BS increased oxidative stress and levels of IL-6, IL-8, TNF-α. Levels of PM_10_ and t,t-MA were 2.9- and 5.8-times higher in biomass-using women.	[91]

Biomass smoke (BS); cigarette smoke (CS); liquefied petroleum gas (LPG); particulate matter with diameter less than 10 and 2.5 micrometers (PM_10_ and PM_2.5_); reactive oxygen species (ROS); superoxide dismutase (SOD); tumor necrosis factor-alpha (TNF-α); interleukin-6, interleukin-12, interleukin 8 (IL-6, IL-12, IL-8); C-reactive protein (CRP); trans, trans-muconic acid (t,t-MA).

**Table 3:** Summary of the in vitro and in vivo biological responses of the human airway epithelium to woodsmoke exposure.

Biological Response of Epithelial Cells		PM-Derived Woodsmoke		Water-Soluble Wood Tar		Organic-Soluble Wood Tar
		In Vitro	In Vivo	In Vitro	In Vivo	In Vitro
Inflammatory cytokines	IL1-β	✓		✓	✓	
	TNF-α	✓		✓	✓	
	IL-8	✓		✓		
	IL-6	✓			✓	
Antioxidant genes	OH-1	✓		✓	✓	X
	MT-2	✓			✓	
	CYP2E	✓			✓	
	Cyp1a1			✓		X
Oxidative stress	Increased superoxide anion production	✓		✓		✓
	Increased MDA levels			✓		
Mitochondrial dysfunction	Decreased MMP	✓		✓		✓
	Increased phospho-DRP1	✓				
	increased mtROS	✓				
	Decreased ATP levels	✓				
	Decreased oxygen consumption	✓		✓		
	Change in morphology	✓				
	Increased swollen mitochondria with depletion of cristae in lung		✓			
Cytotoxicity	Cell death	✓		✓		✓
Genotoxicity	Cell cycle arrest			✓		✓
	DNA damage	✓		✓		✓
Barrier dysfunction	Reduction of E-cadherin, loss of cobblestone patterning	✓				
Increased mucin production	Increased expression and secretion of MUC5AC	✓	✓			
ER stress	Release of Ca^2+^	✓				

Heme oxygenase-1 (HO-1); metallothionein-2 (MT-2); and cytochrome P450 2E (CYP2E).

**Table 4:** Summary of studies on inflammatory markers in biomass smoke-related COPD (BS-COPD).

Analysis	Study Population	Findings	Reference
Serum IL1-β and TNF-α. FEV_1_	BS-COPD, all females, n = 40, biomass exposure ≥ 40 hour-years; CS-COPD, all male, n = 40, smoking >10 pack-years, and healthy controls, 50% female, n = 80. Biomass fuel for cooking, no specific source.	Higher IL1-β and TNF-α in COPD patients vs. controls. Negative linear relationship between TNF-α and IL1-β with FEV1 in COPD patients. Association of TNF-α levels with CS-COPD. Association of IL1-β levels with both CS- and BS-COPD. BS-COPD patients had higher levels of IL1-β but lower levels of TNF-α than CS-COPD	[143]
40 serum chemokines and cytokines by multiplex immunoassay, including IL1-β and TNF-α, IL-6, IL-8	BS-COPD, all females; n = 29; biomass exposure = 112 hour-years; CS-COPD, all male, n = 23, 22.2 pack-years; BS-exposed subjects without COPD (BS-CTRL) all-female, n = 24, biomass exposure = 120 hour-years; and smokers without COPD (CS-CTRL), all male, n = 22; 16.15 pack-years). Only dry firewood as fuel for cooking and heating.	Lower CCL15, CCL27, and CXCL13 in BS-COPD vs. BS-CTRL. Distinct levels of CCL1, -7, -15, 17, -19, -CXCL2, IFNγ, and MIF in CS-COPD vs. CS-CONTROL	[144]
Sputum cellular inflammatory components: total cells, neutrophils, eosinophils, lymphocytes, macrophages.	BS-COPD, n = 28, all females; CS-COPD, n = 85, 79% male. No healthy controls. Biomass fuel for cooking, no specific source.	Sputum eosinophilia in BS-COPD. Neutrophil infiltrate in sputum in both BS-COPD and CS-COPD.	[145]
Blood cell count, CRP, fibrinogen, and IgE. Lung function.	BS-COPD, n = 31, biomass exposure = 340.90 ± 206.09 hour-years; CS-COPD, n = 49, 41.57 ± 25.62 pack-years; CS + BS-COPD patients with both exposures, n = 46, biomass exposure = 345.2 ± 193.2 hour-years, 55.5 ± 47.1 pack-years; and healthy controls (n = 52). Use of indoor open fire with coal, coke, wood, pellet, agricultural residue or animal dung for cooking or heating.	CS + BS-COPD showed worse blood oxygenation by oxygen saturation, spirometry, and diffusing capacity. BS-COPD and CS + BS-COPD showed higher levels of IgE than CS-COPD and controls. CS-COPD showed higher CRP levels than BS-COPD. Fibrinogen was higher in CS-COPD and CS + BS-COPD patients than controls.	[146]
Exhaled nitric oxide, serum IL-6, IL-8, IL-5, IL-13, periostin, SP, TNF-α, IgE, ERS, CRP, and fibrinogen	BS-COPD, n = 20, biomass exposure = 262 hour-years; CS-COPD, n =20, smoking = 10 pack-years; and healthy controls, n =20. Cooking or heating with biomass fuel. No specific source.	All inflammatory markers were higher in both types of COPD compared to healthy controls, except for IL-13, SP, neutrophil %, eosinophil count, ERS, and IgE. Lower levels of IL-6, IL-8, and IL-5 in BS-COPD than CS-COPD	[147]
Serum levels of SCGB1A1. Lung functions.	BS-COPD, n = 50, biomass exposure = 75 hour-years, 78% female; CS-COPD, n = 50, 66 pack-years, all male, CS controls, n = 50, 61 pack-years; and unexposed healthy controls, n = 50. Cooking with biomass fuel, no specific source.	Lower levels of SCGB1A1 in BS-COPD and CS-COPD compared to CS controls and healthy controls. SCGB1A1 levels were positively correlated with FEV1, FVC, and exercise capacity.	[148]
Serum MMP-1, MMP-7, MMP-9, MMP-9/TIMP-1, and CRP	BS-COPD, n = 40, biomass exposure = 230 ± 132 hour-years; CS-COPD, n = 40, smoking = 50 ± 30 pack-years; and healthy unexposed controls, n = 40. All females. Cooking with wood as fuel.	Levels of MMP-1, MMP-7, MMP-9, MMP-9/TIMP-1, and CRP were higher in both BS-COPD and CS-COPD compared to controls.	[149]

**Table 5:** Studies of miRNAs in biomass smoke-related COPD (BS-COPD) compared to cigarette smoke-related COPD (CS-COPD).

Analysis	Study Population	Size Cohort (n)	Findings	Reference
Level of miR-22 in serum by qPCR	BS-COPD and CS-COPD. All women, stages III-IV. Biomass from wood-burning.	25 vs. 25	Downregulated miR-22 in BS-COPD vs. CS-COPD	[159]
Screening: 96 miRNAs from a set of Human PCR Array (Qiagen). Validation: qPCR. Sample: serum	BS-COPD, CS-COPD, stages I-II, all women. Biomass-exposed controls, control smokers, controls without exposure (controls), all women. Biomass from wood-burning.	Screening: 3 samples for each study group. Validation: 25 samples for each study group.	Downregulated miR-34a-5p in BS-COPD vs. CS-COPD. Downregulated miR-374a-5p in BS-COPD vs. controls. Upregulated miR-191-5p in BS-COPD vs. biomass-exposed controls. Downregulated miR-21-5p in CS-COPD vs. controls.	[160]
Screening: 2069 miRNAs in serum by high-throughput sequencing.	Never-smoking BS-COPD, and ever-smokers CS-COPD. 66% female for each group. Organic source of biomass not provided.	15 vs. 15	45 miRNAs differentially expressed in BS-COPD vs. CS-COPD	[162]

Quantitative PCR (qPCR).

**Table 6:** Genetic studies of biomass smoke-related COPD (BS-COPD) compared with biomass-exposed healthy controls (BS-controls) in predominantly female populations.

SNPs	Significant Association with Biomass-Related COPD	*p* Value and Odds Ratio (95% CI)	Size Cohort	Reference
Chr 12: Gene HSP90B1 rs2070908	Decreased risk of COPD	*p* < 0.01, OR = 0.6, 95% CI: (0.4–1.0)	186 BS-COPD vs. 444 BS-controls	[179]
Chr 6: Gene IL17A rs2275913 rs8193036	Risk of COPD	*p* = 4.52 × 10^−17^, OR = 1.11, 95% CI: (1.08–1.13). *p* = 3.15 × 10^−17^, OR = 1.11, 95% CI: (1.084–1.138)	190 BS-COPD vs. 183 BS-controls	[180]
Chr 4q31: Gene HHIP rs13118928 rs13118928–rs1828591	Decreased risk of COPD	*p* = 0.021, OR = 0.51, 95% CI: (0.27–0.97). *p* = 0.04, OR = 0.65, 95% CI: (0.43–0.98)	186 BS-COPD vs. 557 BS-controls	[182]
Chr 14q32: Gene SERPINA1 rs17580 (PiS allele)	Risk of COPD	*p* < 0.0001, OR = 11.46, 95% CI: (3.12–42.03).	98 BS-COPD vs. 275 BS-controls	[191]
Chr 14q32: Gene SERPINA1 rs1303 rs709932	No association	*p* > 0.05	178 BS-COPD vs. 551 BS-controls	[185]
Chr 6: Gene TNF rs1800629, rs361525, and rs1800750	No association	*p* > 0.05	168 BS-COPD vs. 96 BS-controls	[186]

Biomass-related COPD patients and biomass-exposed healthy controls were chronically exposed to wood-burning biomass smoke. Patients and controls were predominantly female (89–99%). Chromosome (Chr); heat shock protein 90 beta family member 1 (HSP90B1); interleukin 17A (IL17A); Hedgehog interacting protein (HHIP); serpin family A member 1 (SERPINA1); tumor necrosis factor (TNF).

**Table 7:** Pathobiology of biomass smoke-related COPD compared to cigarette smoke-related COPD.

BS-COPD	CS-COPD
**PHENOTYPE**	
Less emphysema, more scarring in the lung parenchyma and in the walls of the bronchi, bronchioles, and blood vessels, and greater anthracosis. FEV1 annual rate decline lower than CS-COPD. Mucus overproduction and hypersecretion. Bronchitis.	Severe emphysema, more pronounced goblet cell metaplasia, mucus overproduction and hypersecretion, bronchitis, and accelerated FEV1 annual rate decline.
**SMOKE PATTERN OF INHALATION, CONCENTRATION AND DEPOSIT IN THE LUNG**	
Household air pollution inhaled at normal tidal volume with low flow rates through the nose (nasal breathing) and likely limits the penetration of smoke beyond the small airways resulting in an airway-predominant COPD phenotype.	Deep oral inhalation of cigarette smoke with higher flow rates. Higher concentrations of cigarette smoke and higher and deeper PM intrathoracic depositing in the lungs leading to emphysema.
**MECHANISMS OF PATHOGENESIS**	
**Inflammation**	**Inflammation**
Not conclusive on the type of inflammatory cell components.	Airway inflammation. Bronchial tree and lung tissue infiltrated by neutrophils, macrophages, and lymphocytes
Not conclusive on the type of inflammatory cytokines components.	Increased IL1-β, TNF-α, IL-8, GM-CSF, TGF-β, MCP-1, LTB_4_, CXCL9, CXCL10, and CXCL11.
**Oxidative stress, oxidative damage**	**Oxidative stress, oxidative damage**
Higher plasma levels of MDA and increased activity of SOD.	Excess ROS and reactive nitrogen species generation, antioxidant depletion and reduced antioxidant enzyme activity.
**Protease anti-protease imbalance**	**Protease anti-protease imbalance**
High levels of MMP-1, MMP-7, MMP-9, MMP-9/TIMP-1, and CRP.	Increased proteolytic enzymes MMP-2, MMP-9, MMP-12, and cathepsins. Increased neutrophil-derived protease 3 and elastase. ECM proteolysis. Affectation of airway remodeling
**Cytotoxicity, DNA damage, apoptosis and cell death**	**Cytotoxicity, DNA damage, apoptosis and cell death**
DNA damage.	Cytotoxicity, DNA damage and apoptosis of alveolar cells.
**Mitochondrial dysfunction**	**Mitochondrial dysfunction**
No information available in BS-COPD	CS-induced dysregulation of NLRX1/MAVS
**Senescence**	**Senescence**
No information available in BS-COPD	Association od COPD with characteristics of “aging lung.”
**Genetic susceptibility**	**Genetic susceptibility**
Association between selected SNPs and genetic susceptibility to COPD in nonsmokers, primarily women, chronically exposed to wood-biomass smoke.	FAM13A on 4q22, HHIP on 4q31, IREB2 and nicotinic acetylcholine receptors (CHRNA3 and CHRNA5) on 15q25, the 19q13 locus with genes RAB4B, EGLN2 and CYP2A6, RIN3 on 14q32, MMP12 on 11q22, and TGFB2 on 1q41.
**Epigenetic regulation**	**Epigenetic regulation**
MicroRNAs: preliminary evidence that BS-COPD patients might have a distinct miRNAs expression profile compared to CS-COPD.	MicroRNAs: changes in microRNA profiles of bronchial biopsies, lung tissue, airway cells, sputum, and peripheral blood in COPD patients are linked to disease status, diagnosis, survival, pathogenesis, and response to corticosteroid treatment

Malondialdehyde (MDA); superoxide dismutase (SOD); nucleotide binding domain and leucine-rich-repeat-containing protein X1 (NLRX1); surfactant protein-P (SP); erythrocyte sedimentation rate (ERS); C-reactive protein (CRP); secretoglobin family 1A member 1 (SCGB1A1); matrix metalloproteinase (MMP).

## Data Availability

No new data were created or analyzed in this study. Data sharing does not apply to this article.

## Supplementary Materials

The following supporting information can be downloaded at https://www.mdpi.com/article/10.3390/cells12010067/s1. Table S1: Comparison between the pollutant components present in wood smoke and cigarette smoke.
